# Exploratory analysis of perioperative plasma histamine concentrations in dogs with mast cell tumours

**DOI:** 10.3389/fvets.2026.1882434

**Published:** 2026-09-11

**Authors:** Akihiro Niwa, Tsuyoshi Kadosawa, Toshikazu Sakai

**Affiliations:** Department of Companion Animal Clinical Science, School of Veterinary Medicine, Rakuno Gakuen University, Ebetsu, Hokkaido, Japan

**Keywords:** canine, exploratory, histamine, mastocytoma, prognosis

## Abstract

**Background:**

In this exploratory study, we investigated plasma histamine concentration as a perioperative prognostic marker in dogs with mast cell tumours. Mast cell tumours exhibit marked heterogeneity in biological behaviour, and histological grading is widely used for prognostication; however, grading alone does not directly reflect tumour burden or residual disease following surgery. Histamine has a short half-life in the circulation, and persistently elevated plasma histamine levels may reflect continuous release from neoplastic mast cells. Although plasma histamine concentration has been proposed as a marker of prognosis and residual disease, its clinical relevance and optimal interpretation remain incompletely defined. Twenty-seven dogs that underwent surgical excision of mast cell tumours (29 surgeries) during a 28-month study period were included. Associations between plasma histamine concentration and survival outcomes were evaluated using Kaplan–Meier survival analyses. Multivariable analyses were performed to explore factors associated with preoperative and postoperative plasma histamine concentration.

**Results:**

Dogs with elevated preoperative plasma histamine concentration (>1.0 ng/mL) had significantly shorter progression-free intervals (PFIs) than those with low preoperative plasma histamine concentration (≤1.0 ng/mL). However, in multivariable analyses restricted to dogs with cutaneous mast cell tumours, preoperative plasma histamine concentration was confounded by tumour volume and was not an independent prognostic factor following adjustment. Postoperative plasma histamine concentration values were elevated in only a few cases, limiting formal survival analyses; nonetheless, multivariable analyses demonstrated a significant association between postoperative plasma histamine concentration and gross residual disease, suggesting that postoperative plasma histamine concentration may reflect macroscopic residual tumour but may lack sensitivity for microscopic disease.

**Conclusion:**

The findings suggest that preoperative plasma histamine concentration primarily reflects tumour burden in dogs with cutaneous mast cell tumours, whereas postoperative plasma histamine concentration is associated with macroscopic residual disease and may be less informative for detecting microscopic residual or metastatic disease. Plasma histamine concentration may serve as an adjunctive perioperative biomarker when interpreted in conjunction with established clinicopathological factors. Larger prospective studies are warranted to validate assay performance, refine cut-off values, and determine the clinical utility of plasma histamine concentration in canine mast cell tumours.

## 1 Introduction

Canine mast cell tumours (MCTs) primarily arise in the skin, subcutaneous tissue, spleen, liver, and intestinal tract. The most common presentations are cutaneous and subcutaneous MCTs, which frequently metastasise to regional lymph nodes (LN). The biological behaviour of MCTs ranges widely from benign to highly malignant (1). Histological grading systems, such as those proposed by Patnaik and Kiupel, are the most consistent methods for predicting the biological behaviour of cutaneous MCTs and have been widely used for prognostication (1–3). Although histological grading provides important information regarding tumour aggressiveness, it does not directly assess clinically relevant aspects such as the presence of residual disease after surgery or microscopic metastasis. Consequently, biomarkers that can dynamically and immediately reflect tumour burden remain warranted.

Histamine has a short half-life in the circulation, a characteristic that may be exploited for clinical assessment (4–6). Elevated PHC has been associated with disease progression and poor clinical outcome in dogs with MCTs (5, 6). However, the interpretation of postoperative PHC is less straightforward because postoperative changes may be influenced by surgical and perioperative factors (7). Previous studies have shown that although dogs with allergic skin disease have higher cutaneous histamine levels, their PHCs do not remain elevated and do not strongly correlate with disease severity. The increases are usually limited to transient systemic reactions, such as anaphylaxis (8, 9). In healthy dogs, PHC is typically ≤0.8 ng/mL, whereas dogs with mast cell tumours exhibit significantly higher PHC values (5, 6, 10–13).

Despite these suggested utilities, there has been little progress in research on the clinical significance of PHC in canine MCTs. This may be attributed to the relative robustness of established prognostic factors and PHC’s susceptibility to perioperative influences, including anaesthetic and analgesic drugs and surgical stress, which are difficult to rigorously control in clinical studies. In addition, the heterogeneity of tumour volume, pathological characteristics, and treatment strategies makes it challenging to define uniform study populations.

This study aimed to explore the association between perioperative changes in PHC and prognosis in dogs with MCTs and to assess the potential clinical utility of this association.

## 2 Materials and methods

### 2.1 Ethics approval and consent to participate

This study was approved by the Institutional Animal Care and Use Committee of Rakuno Gakuen University (Ebetsu, Hokkaido, Japan; approval number: 2025-R012; approved on 20 April 2026). Written informed consent for blood sample collection and the use of clinical data for research purposes had been obtained from the owners of all dogs at the time of sample collection.

### 2.2 Participants

In this exploratory study, we investigated PHC as a perioperative prognostic marker in dogs with MCTs.

All dogs with MCTs that underwent surgical excision at a university-affiliated veterinary medical centre (Rakuno Gakuen University) during the study period were eligible for inclusion, and all such cases were consecutively included. This study used clinical data and samples collected as part of routine clinical practise. Written informed consent for blood sample collection and use of clinical data for research purposes was obtained from the owners of all dogs at the time of sample collection. All procedures were performed as part of routine clinical care, and no additional interventions were conducted for research purposes.

Signalment data (age, sex, body weight, and breed), tumour volume (estimated using an ellipsoid approximation and calculated as (π/6) × the maximum tumour diameter × the square of the minimum diameter) (14), histological grade, surgical margin status (complete or incomplete), macroscopic residual disease, regional LN metastasis, progression-free interval (PFI), and survival time were obtained from medical records. PFI was defined as the interval from the date of surgery (day 0) to the detection of disease progression, including local recurrence, metastasis, or the appearance of new macroscopic lesions. Cases with microscopically incomplete surgical margins but no macroscopic residual disease were included in the PFI analysis because no gross disease was detectable immediately after surgery. In contrast, cases with macroscopic residual disease following surgery were excluded from the PFI evaluation because macroscopic disease was already present immediately after surgery, precluding assessment of the interval from surgery to subsequent disease progression. Postoperative gross residual disease was defined as visible residual local tumour remaining at the surgical site after completion of surgery. In the present study, these lesions consisted of unresected satellite lesions surrounding large mast cell tumours. Data from dogs that died without evidence of disease progression were censored at the date of death. Cases lost to follow-up were censored at the date of last confirmed contact. Overall survival (OS) was defined as the interval from surgery to death from any cause, with dogs alive at the last follow-up or lost to follow-up censored at the date of last confirmed contact.

Histological grade, surgical margin status, and the presence of LN metastasis were determined based on pathological findings from the resected primary MCTs and the corresponding regional LN. Sentinel lymph node mapping was not performed; instead, anatomically regional lymph nodes and/or adjacent lymph nodes that were clinically enlarged were excised for evaluation.

Information regarding anaesthetic and analgesic protocols, including the use of drugs potentially affecting plasma histamine concentrations, was extracted from the medical records for all procedures.

### 2.3 Sample preparation and plasma histamine assay

Blood samples were collected before surgery and 12–24 h after surgery into EDTA-containing plastic tubes. Samples were centrifuged at 1,400 × g for 5 min at room temperature, and the plasma supernatant was carefully separated from the buffy coat and stored at −20 °C until analysis. This sample processing protocol was recommended by the commercial laboratory (Fujifilm Monolith Co., Ltd.) that performed the plasma histamine measurements. The plasma samples were subsequently submitted to a commercial laboratory (Fujifilm Monolith Co., Ltd.) for PHC determination.

PHCs were measured by Fujifilm Monolith Co., Ltd. using a commercially available enzyme immunoassay kit (Histamine IM2015, Beckman Coulter/Immunotech, Marseille, France). In this assay, acylated histamine in the samples competitively bound to a monoclonal anti–acylated histamine antibody against enzyme-labelled acylated histamine.

### 2.4 Pathological assessment

Histological grading and surgical margin assessment were performed on resected primary tumour tissues. Histological grade was determined according to the Patnaik grading system (grades I–III) (3) for cutaneous mast cell tumours only. Subcutaneous, mucosal, and visceral mast cell tumours were not graded using this system. In cases with multiple lesions, the highest histological grade was used as the representative grade for analysis. Regional LN were removed concurrently with the excision of the primary MCT. All samples were evaluated by a board-certified veterinary pathologist (Diplomate of the American College of Veterinary Pathologists). Regional lymph nodes were evaluated histopathologically for the presence or absence of metastasis. Because these cases were collected before widespread adoption of the Weishaar classification (15), the original pathology reports did not classify lymph nodes according to HN categories. Based on consultation with the board-certified veterinary pathologist who originally evaluated the cases, lymph nodes reported as metastatic would correspond to HN2 or HN3 under the current Weishaar classification. Surgical margins were classified as complete or incomplete based on the presence of tumour cells at the excision line or deep margin.

### 2.5 Evaluation of PHC

The PHCs were measured preoperatively and postoperatively. Cases were categorised using a cut-off value of 1.0 ng/mL; values >1.0 ng/mL and those ≤1.0 ng/mL were assigned to the high and low PHC groups, respectively. The 1.0 ng/mL cut-off was selected based on previous studies reporting associations between elevated PHCs and adverse clinical outcomes or residual disease in dogs with MCTs (5, 6, 8). For descriptive purposes, perioperative changes in PHC were categorised as a ≥15% decrease, a ≥15% increase, or a change of <15%. The 15% threshold was selected as a pragmatic criterion to distinguish more substantial changes from minor fluctuations and was not intended to represent a validated biological or clinical cutoff.

In addition, PHCs were analysed as a continuous variable to evaluate their associations with clinicopathological parameters, including tumour volume and pathological factors. Dichotomised analyses were performed to facilitate clinical interpretability, whereas continuous-variable analyses were conducted to minimise information loss and to assess potential confounding. Findings from both approaches were interpreted in combination.

### 2.6 Statistical analysis

PFI and OS were analysed to evaluate the prognostic significance of preoperative and postoperative plasma histamine concentrations (PHCs). Survival curves were generated using the Kaplan–Meier method and compared using log-rank tests in Python (version 3.11; Python Software Foundation) with standard scientific libraries (NumPy, SciPy, and Matplotlib). All analyses were independently executed and verified by the authors. Statistical significance was set at *p* < 0.05. For dogs that underwent surgery twice, only data from the first surgery were included in survival analyses. In addition, the association between preoperative PHC as a continuous variable and PFI was explored using univariable Cox proportional hazards regression. Multivariable survival analyses, such as Cox proportional hazards regression, could not be reliably performed because of the limited number of cases and events; therefore, only univariable survival analyses were conducted.

To identify factors associated with preoperative PHC, a multivariable linear regression analysis was performed using preoperative PHC as the dependent variable. This analysis was restricted to dogs with cutaneous MCTs because tumour volume and histological grade could be consistently assessed only in this subgroup. Tumour volume and Patnaik histological grade were included as explanatory variables to determine whether preoperative PHC reflected tumour burden, histological aggressiveness, or both.

To identify factors associated with postoperative PHC, a separate multivariable linear regression analysis was performed using postoperative PHC as the dependent variable. Gross residual disease and regional lymph node (LN) metastasis were included as explanatory variables because both variables were considered potential indicators of residual tumour burden following surgery. Only surgeries in which regional lymph nodes were surgically excised and histopathologically evaluated (*n* = 27) were included in this analysis.

## 3 Results

### 3.1 Animals

Twenty-seven dogs with MCTs (median age: 9.0 years; range, 4–17 years) underwent 29 surgeries. Two dogs each underwent a second surgery for an MCT at a different time point during the study period. The cohort included five castrated males, eight intact males, seven spayed females, and seven intact females. Breeds included Shiba Inu (*n* = 4), Pug (*n* = 2), Toy Poodle (*n* = 2), Miniature Schnauzer (*n* = 2), Bernese Mountain Dog (*n* = 2), American Cocker Spaniel (*n* = 2), Labrador Retriever (*n* = 2), and French Bulldog (*n* = 2); one each of the following breeds was also included: Papillon, Shih Tzu, Golden Retriever, Hokkaido, Jack Russell Terrier, Beagle, Boston Terrier, Pomeranian, and Welsh Corgi. The mean body weight of the dogs was 13.4 kg (range, 3.0–43.8 kg). None of the included dogs was receiving treatment for allergic diseases at the time of surgery. However, three dogs had concurrent chronic kidney disease or myxomatous mitral valve disease. Information regarding medications administered for these concurrent diseases was not consistently available from the retrospective records.

Samples included 16 cutaneous MCTs, 11 subcutaneous MCTs (including 1 scrotal MCT), 1 mucous membrane MCT, and 1 visceral-type MCT. Among the 16 cutaneous MCTs, histological grading according to the Patnaik system was as follows: Grade I, 0; Grade II, 13; and Grade III, 2. One tumour could not be evaluated owing to marked necrosis. Surgical margin assessment revealed 18 complete and 11 incomplete resections; gross residual disease was present in three of the incompletely resected surgeries. Regional LN resection was performed in 27 surgeries, and metastasis was confirmed in 20 cases (74%). The demographic and clinicopathological characteristics of all individual cases are summarised in Supplementary Table S1.

In total, 11 dogs died, of which 8 died from mast cell tumour–related disease and 3 from unrelated causes. Fourteen dogs were alive at the end of the study with a median follow-up duration of 253 days (range, 40–847 days), and two were lost to follow-up. Among the dogs that died, 7 had complete excision and 4 had incomplete excision; among those alive at the end of the study, 9 had complete excision and 5 had incomplete excision. Both dogs lost to follow-up had undergone complete excision. Recurrence was observed in nine dogs (11 surgeries), eight of which died, and one was lost to follow-up. Three dogs died before experiencing recurrence; their causes of death were high-grade lymphoma, acute pancreatitis, and unknown. The dog that died from high-grade lymphoma had no evidence of lymphoma at the time of MCT excision; lymphoma developed several months later, was treated, and subsequently resulted in death. The follow-up duration ranged from 29 to 847 days for the PFI analysis and from 40 to 847 days for the OS analysis.

Five dogs with complete resection and no LN metastasis did not receive additional treatment, except for one dog that underwent intraoperative radiation therapy because adequate surgical margins were considered difficult to achieve. Additional treatments, including molecularly targeted therapy, radiation therapy, chemotherapy, and repeat surgery, were administered variably in high-risk cases (defined as dogs with Patnaik grade III tumours or gross residual disease). Among the remaining 16 dogs with LN metastasis or incomplete resection of cutaneous Grade II or non-cutaneous MCTs, three did not receive additional treatment. The other dogs received one or more adjuvant treatments, including molecular targeted therapy, radiation therapy, chemotherapy, or repeat surgery.

Except for one dog that underwent tumour excision under local anaesthesia, all dogs received sedation followed by induction of general anaesthesia, endotracheal intubation, and maintenance with inhalant anaesthesia. Ketamine and fentanyl were the most commonly used agents for perioperative analgesia.

### 3.2 Perioperative plasma histamine concentrations

**Figure 1:**
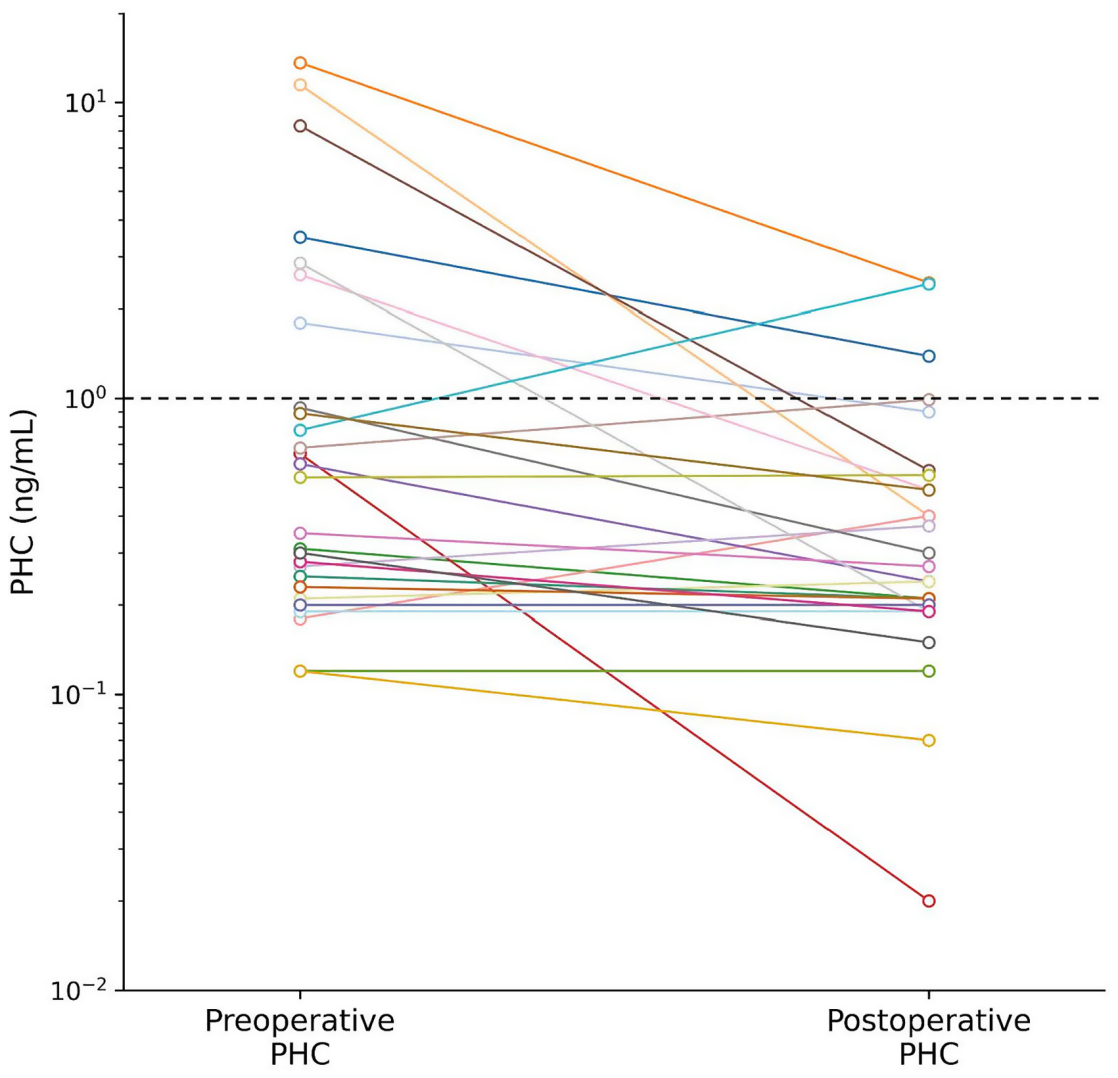
Perioperative dynamics of plasma histamine concentration (PHC). The median preoperative (*n* = 29 surgeries) and postoperative (*n* = 28 surgeries) PHCs were 0.35 ng/mL (range: 0.12–13.7 ng/mL) and 0.26 ng/mL (range: 0.02–2.45 ng/mL), respectively. The dotted line indicates a PHC of 1 ng/mL.

The median preoperative PHC (prePHC) and postoperative PHC (postPHC) were 0.35 ng/mL (range, 0.12–13.7 ng/mL) and 0.26 ng/mL (range, 0.02–2.45 ng/mL), respectively. Perioperative PHC decreased by ≥15% in 17 of 28 surgeries, increased by ≥15% in four, and changed by <15% in seven. Among the four surgeries with increased postPHC, one involved a visceral MCT with histopathologically confirmed macroscopic hepatic lesions and was reclassified from the low PHC group preoperatively to the high PHC group postoperatively. The remaining three surgeries remained in the low PHC group following surgery (Figure 1).

Seven of 29 surgeries were classified into the high prePHC group (>1.0 ng/mL), whereas 22 were classified into the low prePHC group (≤1.0 ng/mL). PostPHC was available for 28 surgeries, of which three were classified into the high postPHC group (>1.0 ng/mL) and 25 into the low postPHC group (≤1.0 ng/mL). Among the three surgeries in the high postPHC group, two also had elevated prePHC (3.50 → 1.39 ng/mL and 13.63 → 2.45 ng/mL), whereas one showed an increase from a low prePHC to an elevated postPHC (0.78 → 2.43 ng/mL).

### 3.3 Association between prePHC and prognosis

**Figure 2:**
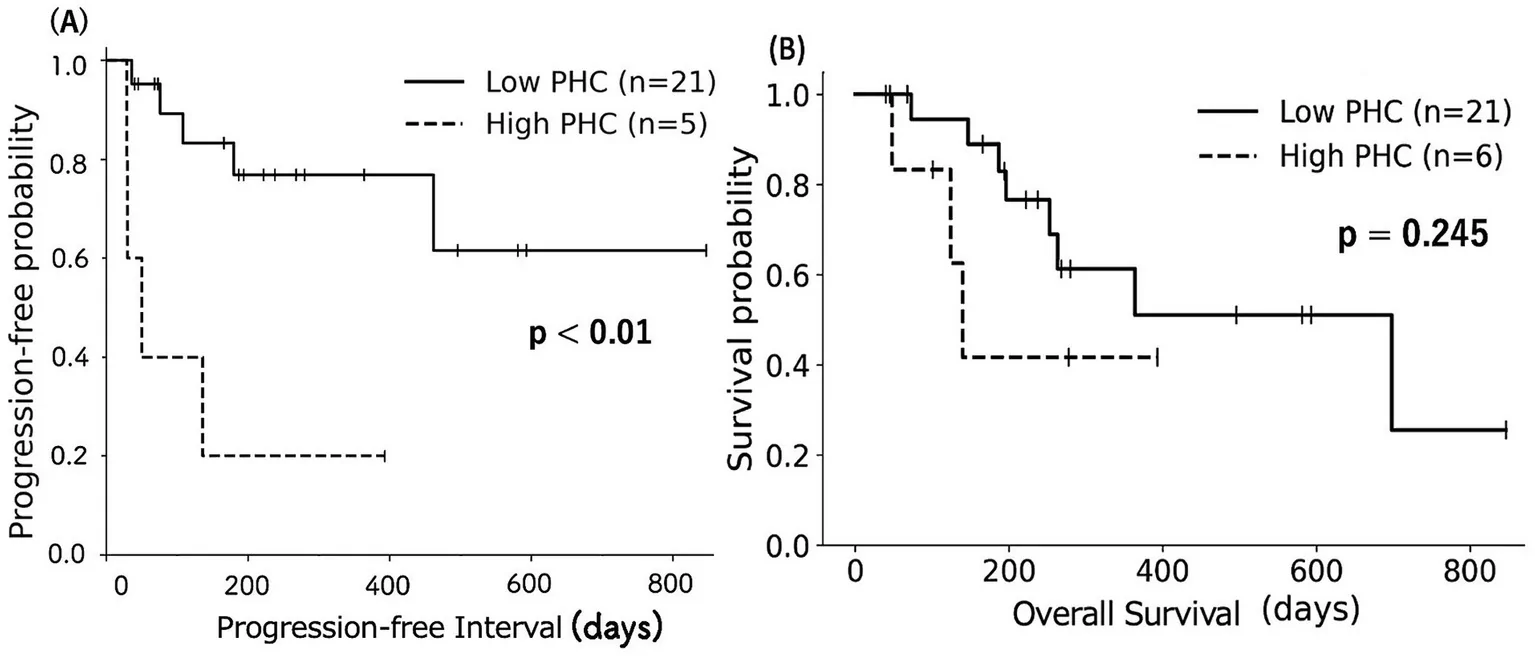
Kaplan–Meier curves for progression-free interval (PFI) and overall survival stratified by preoperative plasma histamine concentration (PHC). **(A)** Kaplan–Meier analysis of PFI stratified according to a preoperative PHC cutoff of 1.0 ng/mL. Preoperative PHC values from 26 surgeries were used to categorise the surgeries into low (*n* = 21) and high (*n* = 5) PHC groups (>1.0 ng/mL, high PHC; ≤1.0 ng/mL, low PHC). A significant association was observed between preoperative PHC and PFI (*p* < 0.01). The median PFI was not reached in the low PHC group but was 50 days in the high PHC group. **(B)** Kaplan–Meier analysis of overall survival stratified according to a preoperative PHC cutoff of 1.0 ng/mL. Preoperative PHC values from 27 dogs were used to categorise the dogs into low (*n* = 21) and high (*n* = 6) PHC groups. For dogs that underwent surgery twice, only data from the first surgery were included. No significant association was observed between preoperative PHC and overall survival (*p* = 0.245). Kaplan–Meier estimated median overall survival times were 698 and 140 days in the low and high PHC groups, respectively. Tick marks indicate censored observations, including dogs alive at the last follow-up and dogs lost to follow-up.

When assessing PFI, three of the 29 surgical procedures with postoperative macroscopic residual disease were excluded because macroscopic disease was already present immediately after surgery, precluding assessment of the interval from surgery to subsequent disease progression, leaving 26 procedures for analysis. Two of the excluded procedures had elevated prePHC, whereas one had low prePHC. PrePHC was significantly associated with PFI (*p* < 0.01). The median PFI was not reached in the low prePHC group, whereas it was 50 days in the high prePHC group (Figure 2A). When analysed as a continuous variable using univariable Cox proportional hazards regression, higher prePHC was significantly associated with a shorter PFI (*p* = 0.004). When assessing survival time, 27 surgical procedures (27 dogs) were included after excluding repeat surgeries. Overall survival (OS) did not differ significantly between the low and high prePHC groups (Figure 2B; *p* = 0.245). Among the 27 dogs included in the analysis, 11 died and 16 were censored. The Kaplan–Meier estimated median overall survival times were 698 days in the low prePHC group and 140 days in the high prePHC group.

### 3.4 Association between postPHC and prognosis

PostPHC was available for 28 of 29 surgeries. Because postPHC measurements were unavailable for one surgery, this case was included in the prePHC analysis but excluded from the postPHC analysis. For the analysis of PFI, three surgeries with postoperative gross residual local disease, consisting of unresected satellite lesions surrounding large mast cell tumours, were excluded, leaving 25 surgeries for analysis. Among these 25 surgeries, disease progression occurred in nine, whereas the remaining 16 were censored during follow-up. Because only three surgeries had elevated postoperative PHC (>1.0 ng/mL), statistical analyses evaluating associations between postoperative PHC and PFI or OS were not performed. The Kaplan–Meier estimated median PFI was 268 days in the low postoperative PHC group, whereas no estimate was provided for the high postoperative PHC group because of the very small sample size (*n* = 3).

### 3.5 Characteristics of surgeries with high PHC

Eight surgeries (seven dogs) showed elevated prePHC and/or postPHC. Only one dog in the high prePHC group survived throughout the observation period (393 days postoperatively) and showed a favourable clinical outcome. This dog had a cutaneous Patnaik grade II MCT with regional lymph node metastasis and microscopically incomplete surgical margins but did not receive any adjuvant treatment. Among the three surgeries classified into the high postPHC group, two involved macroscopic residual MCTs; in these cases, PHC decreased from 13.63 to 2.45 ng/mL and increased from 0.78 to 2.43 ng/mL, respectively, but postPHC remained above the cut-off value in both surgeries. In the remaining surgery, recurrence and regional lymph node metastasis were histologically confirmed 50 days after surgery.

### 3.6 Findings in the low postPHC group

Notably, incomplete surgical margins were present in 8 of 25 surgeries in the low postPHC group (≤1.0 ng/mL). Lymph node metastasis was also detected in 16 of 23 evaluable surgeries within this group.

### 3.7 Multivariable analyses of perioperative PHC

#### 3.7.1 PrePHC

Multivariable analyses were performed to evaluate factors associated with prePHC. Since a validated histologic grading system is available only for cutaneous MCTs, this analysis was restricted to dogs with cutaneous MCTs for which both tumour volume and histologic grade were available.

Tumour volume ranged from <0.1 to 1226.6 cm^3^ (median, 14.1 cm^3^), demonstrating a markedly right-skewed distribution. Therefore, tumour volume was log-transformed before multivariable analysis. In the multivariable model including log-transformed tumour volume and histologic grade (Patnaik grade II vs. III), tumour volume was significantly associated with prePHC, whereas histologic grade was not. In ordinary least squares regression, log-transformed tumour volume showed a significant positive association with log-transformed prePHC (*p* = 0.029), while grade had no significant effect (*p* = 0.648). The overall model did not reach statistical significance (*F* = 3.47, *p* = 0.065).

**Figure 3:**
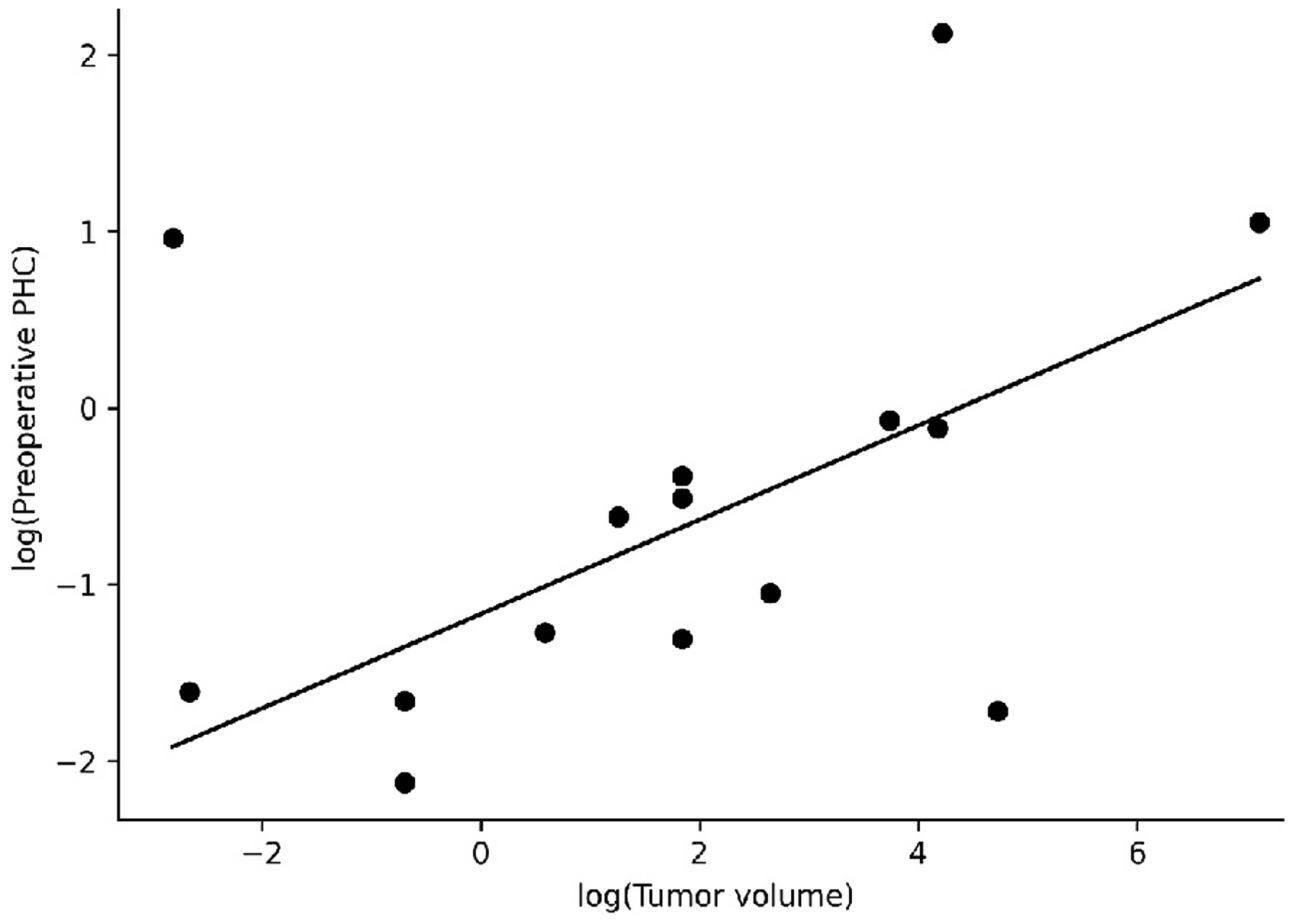
Relationship between tumour volume and preoperative plasma histamine concentration (PHC) in cutaneous mast cell tumours. Scatter plot showing the relationship between log-transformed tumour volume and log-transformed preoperative PHC in dogs with cutaneous mast cell tumours. Each point represents an individual dog. The solid line represents the fitted regression line from robust linear regression analysis. Log-transformed tumour volume was significantly associated with log-transformed preoperative PHC (*β* = 0.27, *p* < 0.001).

To account for the potential influence of outliers, robust linear regression using Huber’s M-estimator was additionally performed. In this model, log-transformed tumour volume remained strongly associated with log-transformed prePHC (*β* ≈ 0.27, *p* < 0.001) (Figure 3), whereas histologic grade again showed no significant association (*p* = 0.654). These findings indicate that prePHC primarily reflects tumour burden rather than Patnaik histologic grade in dogs with cutaneous MCTs.

#### 3.7.2 PostPHC

Multivariable analyses were also conducted to assess factors associated with postPHC. Since postPHC was hypothesised to reflect residual tumour burden rather than tumour subtype, this analysis included dogs with all tumour types. PostPHC values showed no marked right skewness and were therefore analysed on the raw scale. The multivariable model included LN metastasis status (absent vs. present) and gross residual disease. In ordinary least squares regression, the presence of gross residual disease was significantly associated with higher postPHC (*p* = 0.013), whereas LN metastasis was not significantly associated with postPHC (*p* = 0.83).

**Figure 4:**
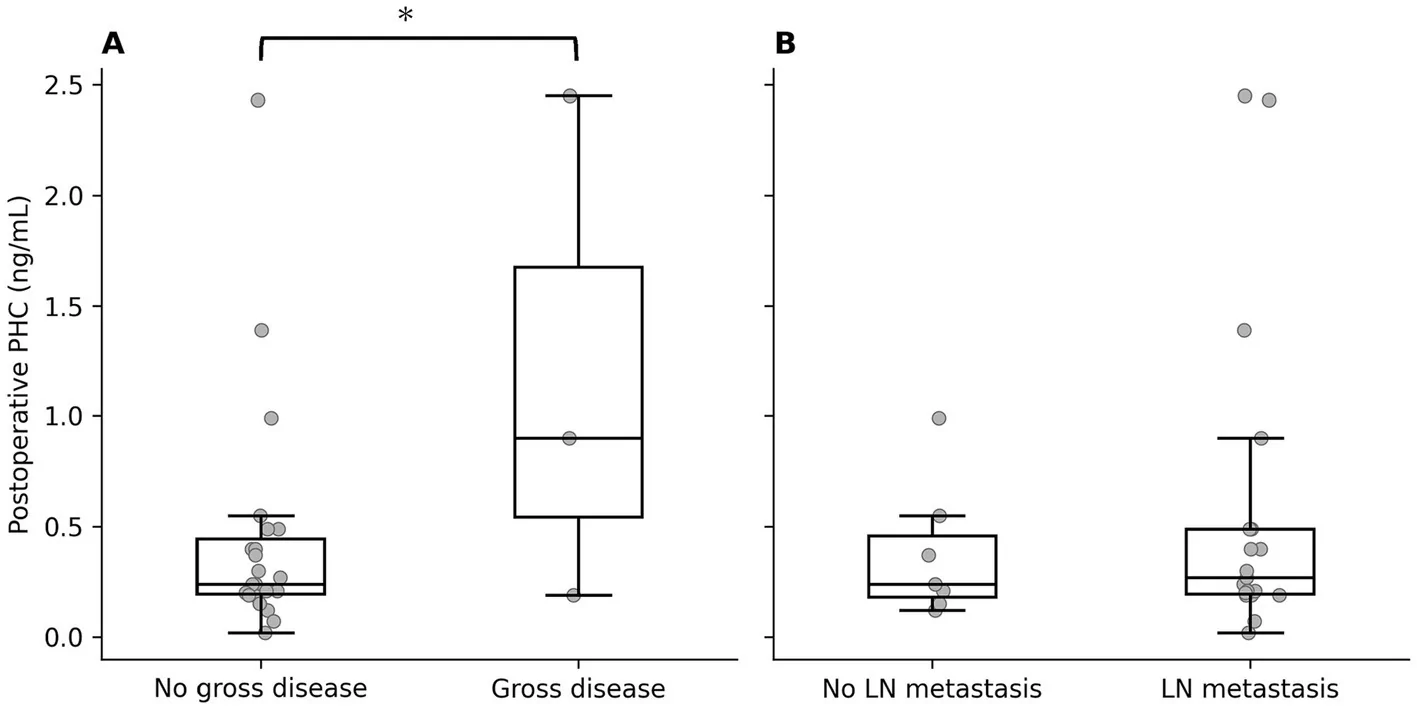
Postoperative plasma histamine concentration (PHC) according to gross residual disease and lymph node (LN) metastasis status. Box-and-whisker plots with overlaid individual data points showing postoperative PHC. Each point represents an individual dog. **(A)** Postoperative PHC according to gross residual disease status. Dogs with gross residual disease had significantly higher postoperative PHC than dogs without gross residual disease. In robust linear regression analysis, the presence of gross residual disease was significantly associated with increased postoperative PHC. ^*^: (*β* ≈ 0.6 ng/mL, *p* < 0.001). **(B)** Postoperative PHC according to LN metastasis status. No significant difference in postoperative PHC was observed between dogs with and without LN metastasis. In robust linear regression analysis, LN metastasis was not significantly associated with postoperative PHC (*β* ≈ −0.01 ng/mL, *p* = 0.96).

Robust linear regression confirmed these findings: dogs with gross residual disease had significantly higher postPHC compared with dogs without gross residual disease (difference of approximately 0.6 ng/mL; *p* < 0.001) (Figure 4), while LN metastasis showed no significant association (*p* = 0.96). These results suggest that early postPHC primarily reflects the presence of macroscopic residual tumour rather than lymph node involvement.

## 4 Discussion

We evaluated the clinical relevance of perioperative PHC in dogs with MCTs, and our results suggest that prePHC and postPHC may reflect distinct biological and clinical aspects of disease status. By integrating survival analyses with multivariable regression models focused on PHC itself, this study provides a framework for interpreting perioperative PHC in the context of tumour burden and residual disease. The present findings suggest that the clinical interpretation of PHC may differ depending on the timing of measurement during perioperative care.

The prognostic association observed for prePHC appears to be largely indirect. Tumour size is an important prognostic factor in canine MCT, with larger tumours consistently associated with poorer outcomes. Previous studies have demonstrated that tumour diameter is an independent predictor of survival and disease progression in dogs with cutaneous MCTs (2, 16). In the present study, prePHC was significantly associated with PFI, with dogs in the high prePHC group exhibiting markedly shorter PFI and survival duration. Although this association should be interpreted with caution because postoperative treatment was not standardised and varied among high-risk dogs, multivariable analyses restricted to dogs with cutaneous MCTs demonstrated that prePHC was primarily associated with tumour volume rather than Patnaik histologic grade. After adjustment for tumour volume, histologic grade was not independently associated with prePHC. This finding is consistent across ordinary least squares regression and robust regression models, indicating that the observed association was not driven by outliers. Taken together, these results suggest that the prognostic association observed for prePHC in univariable analyses may largely reflect its correlation with tumour burden rather than an independent biological effect. Accordingly, prePHC appears to reflect tumour burden rather than intrinsic histologic aggressiveness. This suggests that the apparent prognostic value of prePHC may be largely explained by tumour burden.

In contrast to prePHC, postPHC showed a distinct, potentially more clinically actionable pattern of association. Although formal survival analyses involving postPHC were limited by the small number of cases with elevated postoperative values, multivariable analyses identified a significant association between postPHC and the presence of gross residual disease, whereas LN metastasis showed no independent association. These findings suggest that early postPHC may primarily reflect macroscopic residual tumour at the surgical site rather than nodal involvement or occult metastatic disease. This interpretation is consistent with the observation that markedly elevated postPHC frequently occurred in dogs with gross local residual disease, while dogs with low postPHC also commonly included cases with lymph node metastasis and microscopic incomplete surgical margins. Although the Weishaar histopathological classification was not available for this retrospective cohort, metastatic lymph nodes in the present study would correspond to HN2 or HN3 according to consultation with the board-certified veterinary pathologist who originally evaluated these cases. Furthermore, except for the single visceral MCT case, no dogs were confirmed to have visceral metastasis despite cytological or histopathological evaluation of suspicious hepatic or splenic lesions. Therefore, visceral metastatic disease is unlikely to have substantially influenced the observed association between postoperative PHC and gross residual disease.

The clinical interpretability of postPHC is strongly influenced by the timing of measurement. Previous studies have reported inconsistent postoperative changes in PHC when measurements were obtained shortly after surgery, likely reflecting the effects of perioperative factors such as anaesthesia, analgesic drugs, and surgical manipulation (7). In the present study, PHC was measured 12–24 h post-surgery, a time window at which transient histamine release induced by anaesthetic agents or surgical stimulation is likely to have subsided. This timing may therefore reduce the impact of perioperative confounding factors while allowing detection of histamine release from clinically relevant residual tumour.

Morphine, which has been reported to increase plasma histamine concentrations (12), was not administered in any case in the present study. Nevertheless, anaesthetic and analgesic management was not standardised among patients, and variability in drug selection, dosing regimens, and timing of administration may have influenced perioperative PHC measurements. In addition, complete information regarding medications administered for concurrent diseases was not available, and their potential influence on PHC could not be assessed. This should be considered a limitation when interpreting the observed associations between PHC and clinical variables.

Several other limitations should also be considered. The overall study design was observational, and clinical outcomes were analysed retrospectively. Treatment protocols, including adjuvant therapies, were not standardised and were determined by clinical judgement. Another limitation is that Kiupel grading was not available because it was not routinely reported at the time these cases were evaluated. The original histological specimens were no longer available for all cases, precluding consistent retrospective reassessment according to the Kiupel grading system. Therefore, histologic grade could only be assessed using the Patnaik system, and the possibility that some Patnaik grade II tumours would have been classified as Kiupel high-grade could not be evaluated. Similarly, regional lymph nodes were assessed before widespread adoption of the Weishaar classification. Although lymph nodes classified as metastatic in this study would correspond to HN2 or HN3 based on consultation with the board-certified veterinary pathologist who originally evaluated the cases, distinction between HN2 and HN3 was not possible retrospectively. Furthermore, contemporary comprehensive staging information was not consistently available. Regional lymph nodes were surgically excised in most cases, and suspicious hepatic or splenic lesions were sampled when present; however, systematic cytological staging of regional lymph nodes, liver, and spleen was not performed in every dog. These limitations reduce the ability to fully account for histologic aggressiveness and total tumour burden in the multivariable analyses. Additionally, the relatively small sample size and limited number of events precluded multivariable survival analyses, such as Cox proportional hazards regression, and the small number of dogs with markedly elevated postPHC limited statistical evaluation of its association with survival outcomes. Finally, several limitations related to plasma histamine measurement should be acknowledged. Histamine IM2015, the enzyme immunoassay used in the present study, has previously been used for the determination of plasma histamine concentrations in dogs, including studies involving dogs with mast cell tumours (13). However, formal species-specific analytical validation of this assay for canine plasma has not been fully established. Moreover, a healthy control population from the same geographic region was not included in the present study, precluding direct establishment of a reference interval within the study cohort. The 1 ng/mL threshold used for subgroup analyses was selected as an exploratory cutoff based on previously reported plasma histamine concentrations in healthy dogs and dogs with mast cell tumours and should not be interpreted as a validated clinical threshold. Nevertheless, higher preoperative plasma histamine concentrations remained significantly associated with a shorter progression-free interval when analysed as a continuous variable, suggesting that the observed prognostic association was not solely dependent on the selected cutoff value. However, postoperative treatment was not standardised, and dogs with Grade III tumours or gross residual disease received various additional treatments, including molecularly targeted therapy, radiation therapy, chemotherapy, and repeat surgery. Consequently, differences in postoperative management may have influenced PFI independently of preoperative plasma histamine concentration, and the observed association should therefore be interpreted with caution. Further prospective studies are required to establish reference intervals, validate assay performance in canine plasma, and determine clinically relevant cutoff values for plasma histamine concentration in dogs.

Despite these limitations, the present findings support a cautious but clinically meaningful role for perioperative PHC when interpreted in conjunction with established clinicopathological factors. Furthermore, these results provide a rationale for further prospective studies to optimise clinical application of perioperative PHC in canine MCTs.

## 5 Conclusion

Our results suggest that perioperative PHC may reflect different components of disease status in dogs with MCTs. PrePHC was primarily associated with tumour burden, whereas postPHC was associated with the presence of macroscopic residual disease rather than lymph node involvement. These findings suggest that PHC may have potential as an adjunctive biomarker for perioperative assessment in canine MCTs, although cautious interpretation in conjunction with established prognostic factors is warranted. Larger prospective studies with standardised treatment protocols and predefined statistical analysis plans are warranted to further clarify the prognostic value and optimal clinical application of PHC.

## Data Availability

The original contributions presented in the study are included in the article/Supplementary material, further inquiries can be directed to the corresponding author/s.
